# Supplementary material for: Efficient neural codes naturally emerge through gradient descent learning
Source: Nat Commun. 2022 Dec 29;13:7972. doi: 10.1038/s41467-022-35659-7 (PMC9800366; doi:10.1038/s41467-022-35659-7)
Supplement: Supplementary file 1 — Supplementary Information [file 41467_2022_35659_MOESM1_ESM.pdf]

Supplementary Information

Figures

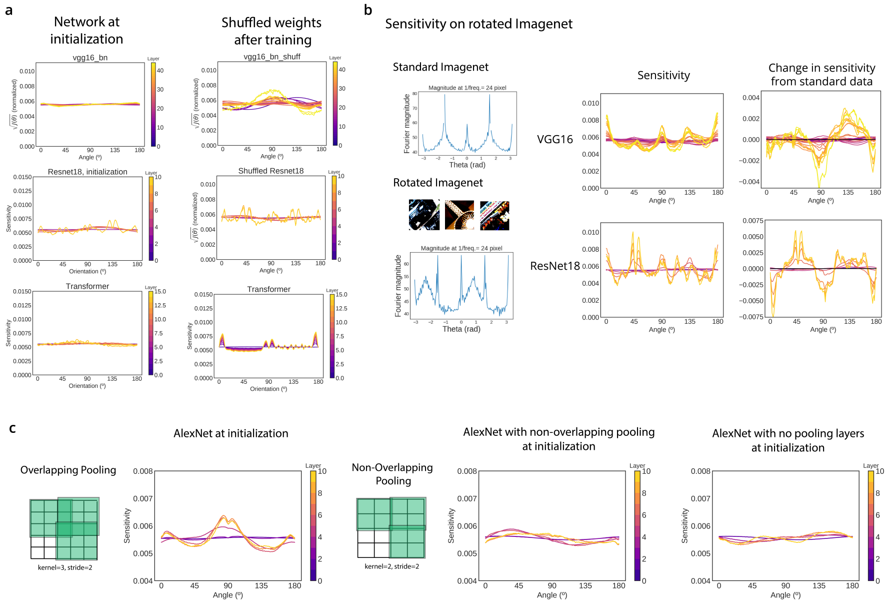

SI Fig 1: Controls for orientation sensitivity analyses. a) The sensitivity to the orientation of Gabor stimuli (see Methods for stimuli parameters) at initialization, left column, and after shuffling the parameters within each layer, right column. b) We retrained the Resnet18 and VGG16 architectures on a version of ImageNet in which all images were rotated by 45º. Left: The orientation statistics can be observed as the magnitude of the Fourier spatial decomposition around a circle centered at the origin in frequency space. This method of analysis will show artifacts of spikes at the cardinals due to the edge effects of rectangular images, but it is a useful control that image statistics do change with rotation. Top is for standard ImageNet, and bottom is for rotated ImageNet. Middle column: The sensitivity of ResNet18 and VGG16 after retraining on rotated images. Though changed, it does not appear as a simple shift of the patterns seen for standard ImageNet (Fig. 2). Right: The difference of this sensitivity pattern from the sensitivity pattern observed after training on standard ImageNet shows that changes do correspond to the change in image statistics, at least in part. c) One source of training-independent sensitivity to the cardinal orientations is overlapping pooling, where it is used. (None of the three above networks employ overlapping pooling.) Left: In AlexNet, which does, the network shows a strong non-uniformity of sensitivity at initialization. Right: The use of non-overlapping pooling greatly diminishes the non-uniformity, as does the complete removal of pooling layers (and accompanying change in the convolutional filter downsampling).

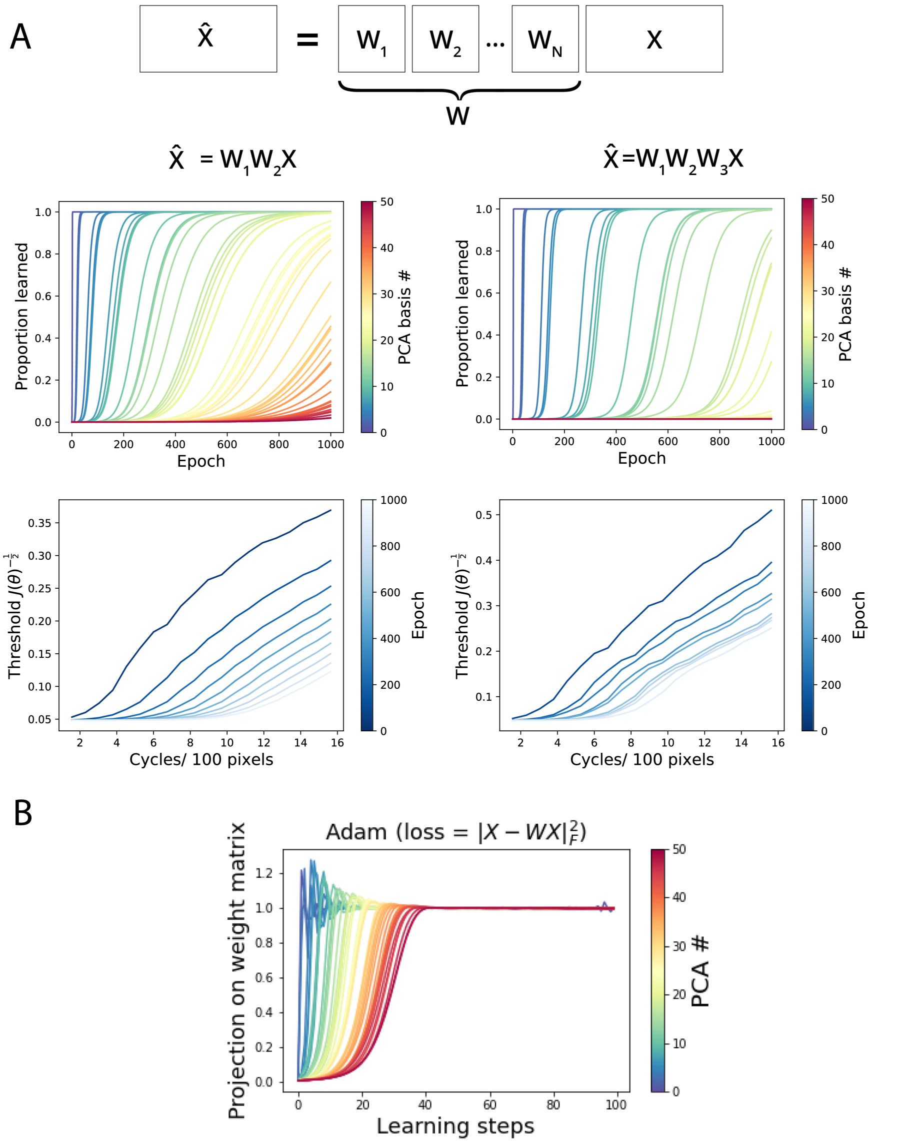

SI Fig. 2: A) Learning dynamics of 2 and 3 layer linear networks. Top: As the depth of networks increases, the principal components are learned in sharper transitions. Bottom: The "threshold" of spatial frequency detection, defined as the inverse square root of the sensitivity to frequency, shows similar patterns regardless of network depth. B) Learning dynamics with the Adam optimizer, instead of gradient descent, for a single-layer linear network. The optimizer is trained with momentum parameters $\beta = (0.9, 0.999)$. Though our theoretical results do not directly apply

to Adam or momentum, it is interesting the degree to which the phenomenon is consistent.

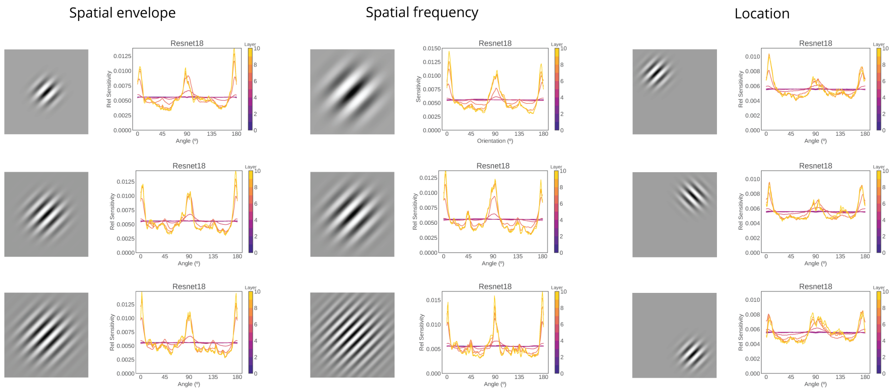

SI Fig. 3: There remains a higher sensitivity to cardinal orientations for different parameterized Gabor functions. The network shown here is the ResNet 18 also shown in Fig. 2. Left column: three Gaussian envelopes of $\sigma = 20, 30$ and 50 pixels and spatial frequency of $f = 4$ cycles per 100 pixels. Middle column: spatial frequencies of $f = 2, 3,$ and 5 cycles per 100 pixels. Right column: three center locations, at $f = 4$ and $\sigma = 2$.

Supplementary Methods

Relation of sensitivity to the Fisher information

In the main manuscript, we define the *sensitivity* of a data point $\mathbf{y}$ to a feature θ as:

$$D(\mathbf{y};\ \theta) = \sum_i \frac{\partial \mathbf{y}_i}{\partial \theta}^2 \tag{1}$$

Throughout this Appendix, lower-case variables represent vectors and capital letters refer to matrices. We denote the sensitivity an ensemble of data points as $D(Y;\ \theta)$.

The Fisher Information

In this section we show that the above definition of sensitivity can be interpreted as the Fisher Information of $\mathbf{y}$ given θ for the case of Gaussian internal noise of unit variance.

Suppose after obtaining $\mathbf{y}$, we observe *noisy* observations of it $\tilde{\mathbf{y}}$. Then, the Fisher information of $\tilde{\mathbf{y}}$ about some input feature θ is:

$$F(\tilde{\mathbf{y}};\ \theta) = -\mathbb{E}_{p(\tilde{\mathbf{y}}|\theta)}\left[\frac{\partial^2}{\partial \theta^2}\log p(\tilde{\mathbf{y}} \mid \theta)\right] \tag{2}$$

When $\mathbf{y}$ is a representation of some inputs x, the Fisher information is that implicitly reflects the stimulus ensemble X, through the connection $\mathbf{x} \to \theta \to \mathbf{y}$. It is often the case that we wish to measure the *average* Fisher information over such an input ensemble. For example, the Fisher Information of orientation on natural images, or on test stimuli. We denote such an average by with capital letters, suggesting a matrix of examples: $F(\tilde{\mathbf{y}};\ \theta)$.

Fisher information for internal noise sources:

We are concerned with the Fisher information $F(\tilde{\mathbf{y}};\ \theta)$ when only a single input $\mathbf{x}$ corresponds to each θ. For example, one could be interested in the Fisher information about orientation as characterized by a set of rotating sinusoidal gratings with identical contrast, phase, and frequency.

Since we observe Y noisily, we still observe a distribution $p(\tilde{\mathbf{y}} \mid \mathbf{x} = f(\theta))$ for each θ. Here we have written $\mathbf{x} = f(\theta)$ for some real function $f : \mathbb{R} \to \mathbb{R}^N$ since $\mathbf{x}$ is deterministic given θ. In this case the Fisher information is

$$F(\tilde{\mathbf{y}} \mid \theta) = -\mathbb{E}_{p(\tilde{\mathbf{y}}|\mathbf{x}=f(\theta))}\left[\frac{\partial^2}{\partial \theta^2}\log p(\tilde{\mathbf{y}} \mid \mathbf{x} = f(\theta))\right] \tag{3}$$

This is the expression we focus on in this paper. It is simple to calculate using derivatives (as detailed below) and a valid point of comparison to human data characterized with a stimulus ensemble with only one θ per $\mathbf{x}$.

The addition of external noise sources:

As an aside, if one wants to determine the Fisher information about orientation *on natural images*, the desired quantity changes. In naturalistic datasets there are many

inputs $\mathbf{x}$ that could correspond to each value of θ. Thus, there are two sources of noise: *external* noise producing the distribution $p(x \mid \theta)$, and *internal* noise producing $p(\tilde{\mathbf{y}} \mid \mathbf{y})$ or alternatively $p(\tilde{\mathbf{y}} \mid \mathbf{x})$. In this case the Fisher information is not easily obtained from derivatives of model representations. This is because this quantity requires marginalizing over $\mathbf{x}$ inside of the Fisher expression:

$$F(\tilde{\mathbf{y}} \mid \theta) = -\mathbb{E}_{p(\tilde{\mathbf{y}}|\mathbf{x})}\left[\frac{\partial^2}{\partial\theta^2}\log \mathbb{E}_{p(\mathbf{x}|\theta)} p(\tilde{\mathbf{y}} \mid \mathbf{x})\right] \tag{4}$$

The expectation inside of the log makes this analytically intractable. Approximations may be useful for rather simple $p(\mathbf{x} \mid \theta)$, but this is prohibitive for naturalistic data. Thus our expression for sensitivity is not comparable to the Fisher information about data with external noise.

Gaussian internal noise

The notion of network sensitivity in our paper can be equated with Fisher Information in the case that one observes a noised $\tilde{\mathbf{y}}$ containing an additive injection of zero-mean Gaussian noise. Thus with noise $\zeta \sim \mathcal{N}(0, 1)$,

$$\tilde{\mathbf{y}} = \mathbf{y} + \zeta$$

In this case $F(\tilde{\mathbf{y}};\ \theta \mid X)$ simplifies. Since the noise is independent over outputs y_i and furthermore Gaussian over each output unit,

$$\log p(\tilde{\mathbf{y}} \mid \mathbf{x}) = \sum_i \log p(\tilde{\mathbf{y}}_i \mid \mathbf{x}) \tag{5}$$

$$\log p(\tilde{\mathbf{y}} \mid \mathbf{x}) = -\sum_i (\tilde{\mathbf{y}}_i - \mathbf{y}_i)^2 - \frac{1}{2}\log 2\pi \tag{6}$$

Taking the derivative and expectation over ζ, we obtain the well-known result for the Fisher of Gaussians:

$$-\mathbb{E}_{p(\tilde{\mathbf{y}}_i|\mathbf{x})}\frac{\partial^2}{\partial\theta^2}\log p(\tilde{\mathbf{y}}_i \mid \mathbf{x}) = \mathbb{E}_\zeta[\sum_i \frac{\partial^2}{\partial\theta^2}(\tilde{\mathbf{y}} - \mathbf{y})_i^2] \tag{7}$$

$$= -\mathbb{E}_\zeta[\sum_i \frac{\partial}{\partial\theta}((\tilde{\mathbf{y}}_i - \mathbf{y})\frac{\partial \mathbf{y}}{\partial\theta})_i] \tag{8}$$

$$= \mathbb{E}_\zeta[\sum_i (\frac{\partial \mathbf{y}}{\partial\theta})_i^2 - \zeta(\frac{\partial^2 \mathbf{y}}{\partial\theta^2}])_i] \tag{9}$$

$$= \sum_i (\frac{\partial \mathbf{y}}{\partial\theta})_i^2 \tag{10}$$

Sensitivity analysis of a linear network

Imagine that we have a linear multilayer neural network in which the weights of layer i are parameterized by W_i. The output of such a network with N layers is:

$$Y = W_N W_{N-1} \dots W_2 W_1 X \tag{11}$$

The product matrix is simply W, and $Y = WX$.

The total sensitivity can be broken up into terms that depend on the decomposition of W. This will be the bridge to a theory of learning.

As a minimal example, let us first examine the case of determining the sensitivity as a function of how much the data aligns with each singular vector of W. That is, our feature θ_j is the dot product of the data with the jth right singular vector of the weight product matrix. As discussed later, for autoencoding cost functions this will align with the principal components of the data after a bit of training, and so this might also be said to be the sensitivity about the jth principal component.

Defining the SVD and feature of interest as,

$$W = USV^T \tag{12}$$

$$\theta_j = V_j^T \mathbf{x} \tag{13}$$

For this feature, the expected sensitivity for an input ensemble $p(\mathbf{x})$ is:

$$D(Y;\ \theta_j) = \mathbb{E}_{p(\mathbf{x})} \sum_i \big(\frac{\partial USV^T x}{\partial V_j^T \mathbf{x}}\big)_i^2 \tag{14}$$

$$= \mathbb{E}_{p(\mathbf{x})} \big(US \frac{\partial V^T \mathbf{x}}{\partial V_j^T \mathbf{x}}\big)^T \big(US \frac{\partial V^T \mathbf{x}}{\partial V_j^T \mathbf{x}}\big) \tag{15}$$

Since $\frac{\partial V^T \mathbf{x}}{\partial V_j^T \mathbf{x}}$ is a one-hot vector that is 1 in the jth row and zero everywhere else, it acts to "pick out" the jth column of US. Note that the expectation over $p(\mathbf{x})$ disappears as well.

$$D(Y;\ \theta_j) = (US)_{:,j}^T (US)_{:,j} \tag{16}$$

$$= \sigma_j U_{:,j}^T U_{:,j} \sigma_j \tag{17}$$

$$= \sigma_j^2 \tag{18}$$

Here $U_{:,j}^T U_{:,j} = 1$ because columns of U are orthonormal. Thus, for when $\theta_j = V_j^T \mathbf{x}$, the sensitivity $D(Y;\ \theta)_j$ is constant and is the square of the associated singular value.

More general $\boldsymbol{\theta}$

More general θ can be understood using the result of the last section. The approach is to decompose an arbitrary derivative into the derivatives with respect to right singular vectors, as such: $\frac{\partial \mathbf{y}_\mu}{\partial \theta} = \frac{\partial \mathbf{y}_\mu}{\partial V^T \mathbf{x}}^T \frac{\partial V^T \mathbf{x}}{\partial \theta}$. In this case,

$$D(\mathbf{y};\ \theta) = \frac{\partial W\mathbf{x}}{\partial\theta}^T \frac{\partial W\mathbf{x}}{\partial\theta} \tag{19}$$

$$= (\frac{\partial USV^T\mathbf{x}}{\partial V^T\mathbf{x}}^T \frac{\partial V^T\mathbf{x}}{\partial\theta})^T (\frac{\partial USV^T\mathbf{x}}{\partial V^T x}^T \frac{\partial V^T\mathbf{x}}{\partial\theta}) \tag{20}$$

$$= \frac{\partial V^T\mathbf{x}}{\partial\theta}^T SU^T US \frac{\partial V^T\mathbf{x}}{\partial\theta} \tag{21}$$

$$= \frac{\partial V^T\mathbf{x}}{\partial\theta}^T S^2 \frac{\partial V^T\mathbf{x}}{\partial\theta} \tag{22}$$

$$= \sum_j \sigma_j^2 \frac{\partial V_j^T\mathbf{x}}{\partial\theta}^2 \tag{23}$$

Thus, for arbitrary θ, the sensitivity depends on the derivative of the jth right singular vector with respect to θ times the size of its associated singular value.

The behavior of the singular values

Let us now establish a cost function upon the product matrix.

$$\ell(W_N W_{N-1} \dots W_2 W_1) \tag{24}$$

Results of previous literature

Though many papers have adopted this framework, as cited in the main text, here we quote the result of [1].

Summary:

*During gradient descent, under certain restrictive conditions on the initial values of W_i, the **singular values** of the product matrix evolve qualitatively differently for $N = 1$ vs. $N > 1$. For $N > 1$ they grow larger sigmoidally (roughly one-at-a-time) and in order of their contribution to the cost $\ell(W)$.*

The results to follow examine what happens when we train W_i via gradient descent to minimize $\ell(W)$. Each W_i now becomes a function of time, $W_i(t)$, and

$$\dot{W}_i(t) = -\frac{\partial}{\partial W_i}\ell(W_N W_{N-1} \dots W_2 W_1) \tag{25}$$

In addition we assume that the matrices are initialized in a *balanced* manner, meaning that for all $j < N$,

$$W_{j+1}^T(0) W_{j+1}(0) = W_j(0) W_j^T(0) \tag{26}$$

This holds approximately when the weights are initialized very close to zero.

Lemma (Arora et al. 2019)

The product matrix W(t) can be expressed as:

$$W(t) = U(t)S(t)V^T(t) \tag{27}$$

where $U(t)$ and $V(t)$ have orthonormal columns and $S(t)$ is diagonal

Our theory hinges on the behavior of the diagonal elements of $S(t)$, which we will denote as $\sigma_i(t)$.

Theorem 1 (Arora et al. 2019)

The singular values of the product matrix W (t) evolve by:

$$\dot{\sigma}_i(t) = -N\sigma_i(t)^{\frac{2(N-1)}{N}} \left\langle \nabla_W \ell(W(t)), u_i(t)v_i^T(t) \right\rangle \tag{28}$$

$$= -N\sigma_i(t)^{\frac{2(N-1)}{N}} u_i^T(t)\nabla_W \ell(W(t))v_i(t) \tag{29}$$

Thus, each singular value evolves as a product of a function its current size and the network depth ($N\sigma_i(t)^{\frac{2(N-1)}{N}}$) multiplied by how much the gradient correlates with the rank-1 matrix implied by the singular vectors. Note that if $N = 1$ there is no dependence on the current size of $\sigma_i(t)$.

Another important result concerns the rotation of the unit vectors $u(t)$ and $v(t)$. It states that the vectors are static when they align with the singular vectors of $\nabla \ell W(t)$.

Theorem 2 (Arora et al. 2019)

Assume that at initialization, the singular values of the product matrix W (t) are distinct from zero, and that the matrix factorization is non-degenerate, i.e. has depth $N \geq 2$. Then, for any time t such that the singular vectors of the product matrix W (t) are stationary, i.e. $\dot{U}(t) = 0$ and $\dot{V}(t) = 0$, then $U^T(t)\nabla\ell(W(t))V(t)$ is diagonal.

Relation of input statistics to sensitivity

Our approach is to show that frequency $p(\theta)$ reflects in the covariance of θ. This in turn affects the rate of learning of the singular values of the weight matrix W, at least for certain objectives. This connects the sensitivity, or Fisher Information of $\tilde{\mathbf{y}}$, back to the frequency.

The data covariance affects the learning of $\sigma_i(t)$: autoencoding objective

The base case of our study is the autoencoding objective defined for a set of inputs X:

$$\ell(W) = \frac{1}{N}\sum_i^N (\mathbf{x}_i - W\mathbf{x}_i)^T(\mathbf{x}_i - W\mathbf{x}_i) \tag{30}$$

Our goal is to determine the evolution of $\sigma_i(t)$ that results from this cost function. First, see that

$$\nabla_W \ell(W(t)) = -\frac{1}{N}\sum_i^N (\mathbf{x}_i - W\mathbf{x}_i)\mathbf{x}_i^T \tag{31}$$

$$= -\frac{1}{N}\sum_i^N \mathbf{x}_i\mathbf{x}_i^T + W\frac{1}{N}\sum_i^N \mathbf{x}_i\mathbf{x}_i^T \tag{32}$$

$$= W\Sigma - \Sigma \tag{33}$$

Here Σ is the data covariance, assuming $\mathbf{x}$ is centered.

In general, the time evolution of each singular value $\sigma_i(t)$ is complicated to calculate because the singular vectors can potentially rotate, i.e. $\dot{(U)}(t) \neq 0$. However, for the sake of analysis we can examine a limited case when the direction of the singular vectors is static. This will allow us to obtain an analytic expression for the evolution of the singular values in terms of the data covariance. In particular we will examine the case when the weight matrix is initialized to share right singular vectors (but not singular values) with the data covariance.

By plugging the expression for $\nabla_W \ell(W(t))$ into Theorem 2, it can be seen that if $\Sigma = V\Lambda V^T$ and $W = VSV^T$ for the same V, then

$$U^T(t)\nabla\ell W(t)V(t) = U^T(t)\nabla W(t)\Sigma - \Sigma V(t) \tag{34}$$

$$= U^T(t)U(t)S(t)V^T(t)V(t)\Lambda V^T(t) - V(t)\Lambda V^T(t)V(t) \tag{35}$$

$$= S(t)\Lambda - \Lambda \tag{36}$$

This is diagonal, and thus by Theorem 2 $\dot{V}(t) = 0$ during gradient descent on the autoencoding objective.

By Theorem 1, this initialization results in:

$$\dot{\sigma}_i(t) = -N\sigma_i(t)^{\frac{2(N-1)}{N}}(\sigma_i(t)\lambda_i - \lambda_i) \tag{37}$$

$$= N\lambda_i\sigma_i(t)^{\frac{2(N-1)}{N}}(1 - \sigma_i(t)) \tag{38}$$

Extension to supervised learning

A more general class of objective functions is when $W\mathbf{x}$ is trained to match some target $\mathbf{y}$. The input statistics are again relevant here. If we again take the mean-squared error as the objective,

$$\ell(W) = \sum_j (\mathbf{y}_j - W\mathbf{x}_j)^2 \tag{39}$$

The evolution of the singular values is determined by the gradient,

$$\nabla_W \ell(W) = \sum_j (\mathbf{y} - W\mathbf{x})\mathbf{x}^T \tag{40}$$

$$= W\sum_j \mathbf{x}_j\mathbf{x}_j^T - \sum_j \mathbf{y}_j\mathbf{x}_j^T \tag{41}$$

$$= W\Sigma_{\mathbf{xx}} - \Sigma_{\mathbf{xy}} \tag{42}$$

By Theorem 1, then, we have that,

$$\sigma_i(t) = -N\sigma_i(t)^{\frac{2(N-1)}{N}} u_i^T(t)(W(t)\Sigma_{\mathbf{xx}} - \Sigma_{\mathbf{xy}})v_i(t) \tag{43}$$

$$= -N\sigma_i(t)^{\frac{2(N-1)}{N}} u_i^T(t)W(t)\Sigma_{\mathbf{xx}}v_i(t) + N\sigma_i(t)^{\frac{2(N-1)}{N}} u_i^T(t)\Sigma_{\mathbf{xy}}v_i(t) \tag{44}$$

Thus, the evolution of the singular values depends on two additive terms. One of these (left) has no dependence on the labels $\mathbf{y}$, only on the statistics of the data.

As before, we can gain intuition about this evolution by beginning from an initialization that is axis-aligned with the final solution. For the supervised case, these initializations share the singular vectors of the data/labels, but can differ in the singular values. Given $\Sigma_{\mathbf{xx}} = V\Lambda V^T$ and $\Sigma_{\mathbf{xy}} = UTV^T$, we set $W(0) = USV^T$ for the same U and V. This means that,

$$U^T(t)\nabla\ell W(t)V(t) = S(t)\Lambda - T \tag{45}$$

This is a diagonal matrix, and thus a fixed point of learning.

For this initialization, then,

$$\dot{\sigma}_i(t) = -N\sigma_i(t)^{\frac{2(N-1)}{N}}(\sigma_i(t)\lambda_i - t_i) \tag{46}$$

$$= \lambda_i N\sigma_i(t)^{\frac{2N-1}{N}}(\frac{t_i}{\lambda_i} - \sigma_i(t)) \tag{47}$$

References

[1] Arora, S., Cohen, N., Hu, W., Luo, Y.: Implicit regularization in deep matrix factorization. Advances in Neural Information Processing Systems **32**, 7413–7424 (2019)
